# Free-Living Energy Balance Behaviors Are Associated With Greater Weight Loss During a Weight Loss Program

**DOI:** 10.3389/fnut.2021.688295

**Published:** 2021-09-14

**Authors:** Anna Myers, Diana Camidge, Fiona Croden, Catherine Gibbons, R. James Stubbs, John Blundell, Graham Finlayson, Nicola Buckland

**Affiliations:** ^1^Sport and Physical Activity Research Center, College of Health, Wellbeing, and Life Sciences, Sheffield Hallam University, Sheffield, United Kingdom; ^2^Faculty of Medicine and Health, Appetite Control and Energy Balance Research, School of Psychology, University of Leeds, Leeds, United Kingdom; ^3^Department of Psychology, University of Sheffield, Sheffield, United Kingdom

**Keywords:** weight loss, energy balance, appetite, energy intake, free-living physical activity

## Abstract

**Introduction:** Free-living movement (physical activity [PA] and sedentary behavior [SB]) and eating behaviors (energy intake [EI] and food choice) affect energy balance and therefore have the potential to influence weight loss (WL). This study explored whether free-living movement and/or eating behaviors measured early (week 3) in a 14-week WL programme or their change during the intervention are associated with WL in women.

**Methods:** In the study, 80 women (*M* ± *SD* age: 42.0 ± 12.4 years) with overweight or obesity [body mass index (BMI): 34.08 ± 3.62 kg/m^2^] completed a 14 week WL program focused primarily on diet (commercial or self-led). Body mass (BM) was measured at baseline, and again during week 2 and 14 along with body composition. Free-living movement (SenseWear Armband) and eating behavior (weighed food diaries) were measured for 1 week during week 3 and 12. Hierarchical multiple regression analyses examined whether early and early-late change in free-living movement and eating behavior were associated with WL. The differences in behavior between clinically significant weight losers (CWL; ≥5% WL) and non-clinically significant weight losers (NWL; ≤ 3% WL) were compared.

**Results:** The energy density of food consumed [β = 0.45, *p* < 0.001] and vigorous PA [β = −0.30, *p* < 0.001] early in the intervention (regression model 1) and early-late change in light PA [β = −0.81 *p* < 0.001], moderate PA [β = −1.17 *p* < 0.001], vigorous PA [β = −0.49, *p* < 0.001], total energy expenditure (EE) [β = 1.84, *p* < 0.001], and energy density of food consumed [β = 0.27, *p* = 0.01] (regression model 2) significantly predicted percentage change in BM. Early in the intervention, CWL consumed less energy dense foods than NWL [*p* = 0.03]. CWL showed a small but significant increase in vigorous PA, whereas NWL showed a slight decrease in PA [*p* = 0.04].

**Conclusion:** Both early and early-late change in free-living movement and eating behaviors during a 14 week WL program are predictors of WL. These findings demonstrate that specific behaviors that contribute to greater EE (e.g., vigorous PA) and lower EI (e.g., less energy-dense foods) are related to greater WL outcomes. Interventions targeting these behaviors can be expected to increase the effectiveness of WL programs.

## Introduction

Obesity is a global public health concern in both developed and developing countries affecting over 1.9 billion adults worldwide (1). In England, the rate of obesity has almost doubled in the past 20 years with 63% of adults being classified as overweight or obese in 2018 (2). Overweight and obesity increase the risk of developing life-limiting conditions, such as cancer, cardiovascular disease, and type II diabetes, and can significantly reduce quality of life (3, 4). Weight management strategies are required to combat the obesity epidemic (5); however, for many, weight loss (WL) is difficult and weight regain following successful WL is common (6). Less than 20% of individuals with overweight or obesity were able to maintain a body mass (BM) reduction of 10% after 1 year (7). As such, there is a need to understand energy balance behaviors that influence successful WL.

Globally, 42% of adults report engaging in weight management attempts, with higher prevalence in Europe/Central Asia (61.3%) and in individuals with overweight/obesity and in women (8). The most commonly reported WL strategies were dieting and exercise (8, 9). Despite widespread efforts to lose weight, trends in overweight and obesity levels continue to rise. A large proportion of individuals with overweight and obesity find it difficult to achieve WL through lifestyle interventions (e.g., diet and/or exercise) alone (10). In a systematic review and meta-analysis of commercial WL diets, McEvedy et al. (11) found that 57% of individuals who commenced WL programs lost <5% of their initial BM when intention-to-treat data were analyzed. Even among those who completed the WL programs, 37% lost <5% of their initial BM. Achieving ≥5% WL is considered to be clinically significant because this amount of WL for individuals with a body mass index (BMI) of >25 kg/m^2^ is associated with numerous health benefits, such as reduced blood pressure, cholesterol, and blood glucose which, in turn, reduces the risk of long-term conditions such as cardiovascular disease (12).

Studies have shown that individuals respond differently to WL interventions. The WL response can vary considerably between individuals to the same diet (13) or exercise (14, 15) intervention. Exploring the factors that are associated with clinically (≥5%) and non-clinically significant amounts of WL can help identify predictors of WL success and lead to the development of more effective WL strategies. Previous research has identified several psychological, physiological, and behavioral characteristics associated with WL success (16). These include baseline BM, early WL (17, 18), intervention adherence (19, 20), eating behavior traits, such as Three-Factor Eating Questionnaire Hunger, Disinhibition, and Restraint (21), appetite sensations (22), appetite-regulating hormones (23), fat consumption (24), exercise self-efficacy (25), resting energy expenditure (26), and physical activity (PA) (27).

Less research has explored whether energy balance behaviors, both at baseline and change from baseline, are predictive of WL success (28). Two behavioral components are integral to the energy balance equation: movement behaviors (PA and sedentary behavior [SB]) and eating behaviors (energy intake [EI] and food choice which is reflected in the macronutrient composition of the diet). These behaviors affect energy balance and therefore have the potential to influence WL, yet their association with the degree of WL success has received little attention. The key issue is that objective and accurate measures of energy balance behaviors, particularly in WL situations, are relatively hard to achieve. The identification of pre-existing energy balance behaviors and changes in those behaviors during the intervention that is predictive of WL success could inform personalized WL strategies for those in need of additional support.

The purpose of this study was to assess (i) the relationship among free-living movement behaviors, eating behaviors, and change in BM, (ii) whether free-living movement and eating behaviors existing at the beginning of the intervention, and their change during the intervention, significantly predict WL, and (iii) whether there were significant differences in free-living movement and eating behaviors between those who achieved clinically significant WL (≥5%) and those who did not (≤3%).

## Methods

### Participants

The study was conducted as a secondary analysis of data collected from a trial that is published in more detail elsewhere (29) (ClinicalTrials.gov #NCT02012426). The secondary analysis study protocol was pre-registered on Open Science Framework (https://osf.io/fptwn). Based on the recommendations for the minimum sample size for detecting relationships between variables (30) and previous research (31), it was estimated that 61–84 participants would be sufficient to assess the overall relationships among free-living movement behaviors, eating behaviors, and change in BM (estimated *r* = 0.30–0.35, 0.8 power, and 0.05 alpha). Women with overweight or obesity were recruited by advertisement from the University of Leeds. The inclusion criteria were the following: provided written informed consent, healthy women, aged 18–65 years, BMI between 28 and 45 kg/m^2^, reporting an interest in weight loss and not actively participating in a commercial WL program, not increased PA levels in the past 4 weeks, able to eat most everyday foods, fruits, and vegetables. The exclusion criteria were the following: significant health problems, taking any medication or supplements known to affect appetite or weight, pregnant, planning to become pregnant or breastfeeding, history of anaphylaxis to food, known food allergies or food intolerance, smokers and those who have recently ceased smoking (within the last 3 months), participants receiving systemic or local treatment likely to interfere with evaluation of the study parameters, those who have taken part in a commercial WL program in the last 2 months, individuals who work in appetite or feeding-related areas, unable to consume foods used in the study, individuals who have had bariatric surgery, history of an eating disorder, presence of untreated hypothyroidism, and insufficient English language skills to complete the study questionnaires.

### Design

**Figure 1:**
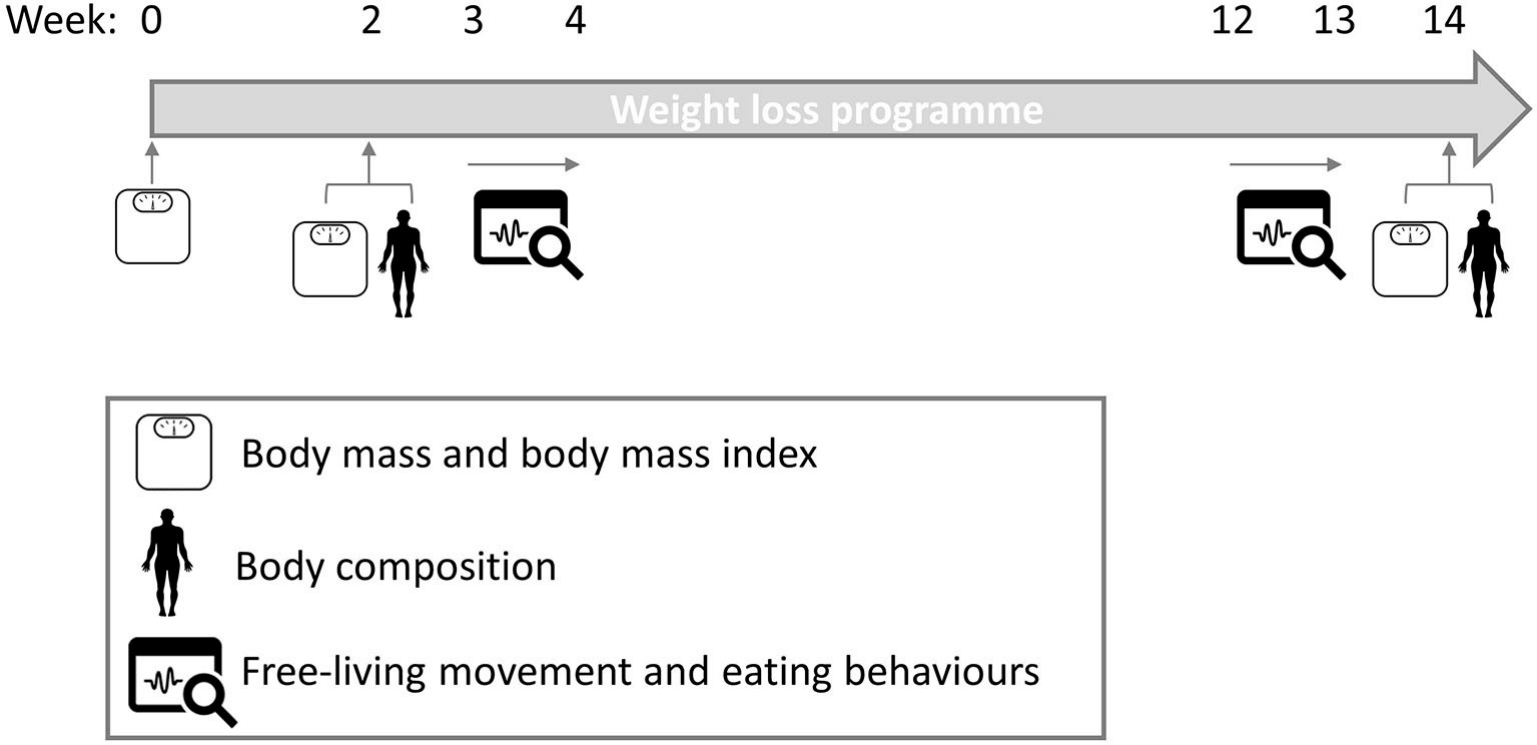
Schematic diagram of study design.

The study was a non-randomized, parallel-group design to assess whether behavioral characteristics (early and early-late change) were associated with ≥5% (clinically significant WL) or ≤3% (not clinically significant WL) WL following a 14 week WL program focused primarily on diet. The trial started with a 2 week run-in period followed by 12 weeks of trial monitoring. The purpose of the run-in period was to ensure the uptake and commitment of participants toward the programs. Figure 1 provides a schematic overview of the study design. Self-reported compliance to the WL program was assessed each week by responding to “How well have you managed to stick with the weight control programme?” on a 100 mm visual analog scale (VAS) anchored at each end with “Not at all well” and “Very well”. During weeks 2 and 14, participants visited the laboratory in the Human Appetite Research Unit at the University of Leeds for measurement days. For all laboratory visits, participants were instructed to fast from 10:00 pm the previous night and to abstain from strenuous exercise and alcohol consumption for at least 24 h before. Compliance was checked on arrival via self-report. The participants received payment of £250 on the completion of the study to reimburse them for their time and expenses. The study procedures and all study materials were reviewed and approved by the School of Psychology Research Ethics Committee at the University of Leeds (14-0090). The study was conducted between September 2014 and December 2015.

### Weight Management Program

The participants either followed a commercial program [Slimming World (32)] or a self-led program [NHS Choices (33)] for the 14 week intervention period. The commercial program encouraged *ad libitum* intake of low energy-dense foods as part of a balanced diet, with weekly weigh-ins, group support, WL goals, and access to online support. The self-led program group accessed free online resources and a self-led diet program that recommends reducing calorie intake by 600 kcal/day. Both the programs encouraged individuals to self-monitor and increase engagement in PA by gradually increasing moderate-intensity aerobic exercise and resistance exercise to meet the Chief Medical Officer PA guidelines of 150 min/week of moderate-intensity PA. The effects of the different programs on BM have been reported previously (29).

For this secondary analysis, there were no specific research questions pertaining to the type of program used to induce WL. Rather, the study sought to assess behavioral characteristics associated with the degree of WL success. As such, data were analyzed with both WL program groups combined (commercial and self-led), and program type was controlled for in all analyses.

### Body Mass, Body Mass Index, and Body Composition

*Baseline (week 0)*: BM was measured on the first day of the weight management program with the use of electronic scales (commercial program group: recorded as part of their first weigh-in at a support group meeting; self-led group: recorded by a researcher at the research unit). Weight was measured with shoes and heavy clothing removed and height was measured using a stadiometer (Seca Ltd., Birmingham, UK) without shoes.

*Week 2 and 14*: BM and body composition (fat mass [FM] and fat-free mass [FFM]) were measured using the BOD POD (Body Composition Tracking System, Life Measurement, Inc., Concord, CA, USA), which uses air displacement plethysmography (34). The participants wore tight clothing and a swim cap to allow for an accurate measure of body volume.

### Free-Living Eating Behavior

Participants completed a 7 day weighed food diary during weeks 3 and 12 (35). Electronic scales and training were provided to ensure detailed descriptions (e.g., brands) and consumed weights of foods and beverages were reported. Total EI, macronutrient composition, and energy density were calculated from the food diary. Energy density was calculated from the contribution of all food and milk (excluded all other drinks) (total EI divided by total weight intake) based on criteria previously used (36). Data were analyzed using a computerized food composition database called WISP 4.0 (Tinuviel Software 2013).

### Free-Living Movement Behavior

Free-living PA and SB were measured during weeks 3 and 12 using the SenseWear Armband mini (SWA; BodyMedia, Inc., Pittsburgh, PA, USA), as has previously been described (37). The participants were instructed to wear the SWA on the posterior surface of their upper non-dominant arm for a minimum of 22 h/day for ≥6 days (except for the time spent showering, bathing, or swimming). For the SWA data to be valid ≥22 h of data per day had to be recorded for at least 5 days (midnight to midnight) including at least 1 weekend day. SB was classified as ≤1.5 metabolic equivalents (METs), light PA 1.6–2.9 METs, moderate PA 3–5.9 METs, and vigorous PA ≥6 METs (38).

### Classification of Clinically Significant and Non-Clinically Significant Weight Losers

To identify differences that could account for individual variability in weight loss, the participants were grouped based on their BM change between baseline (week 0) and week 14. The participants were classified as clinically significant weight losers (CWL; *n* = 41) if they lost ≥5% and non-clinically significant weight losers (NWL; *n* = 33) if they lost ≤3% of their initial BM (18). Those who lost 3.1–4.9% of their initial BM (*n* = 6) were excluded from the analyses leaving two groups that exhibited a different weight loss response.

### Change in Body Mass and Free-Living Movement and Eating Behaviors

Week two BM and BMI and week three free-living movement and eating behaviors will be referred to as “early” and week 14 BM and BMI and week 12 free-living movement and eating behaviors will be referred to as “late”. “Early-late change” in free-living movement and eating behaviors refers to the difference between week 3 and week 12 measures.

### Statistical Analysis

Data are reported as *M* ± *SD* (95% *CI*: lower, upper) throughout unless otherwise stated. All the variables were checked for outliers and normality was assessed using Shapiro-Wilk's test. The missing data were imputed using the last observation carried forward (LOCF) method (39). The analyses were conducted on the participants who completed the trial (completer analyses) and on an intention-to-treat basis (LOCF).

In this study, analysis of covariance (ANCOVA) controlling for the program type were performed to determine whether there was a significant change in baseline, early, and late measures of BM or BMI; or in early and late total daily free-living PA, SB, energy intake, macronutrient intake, or energy density. To explore the relationship among free-living movement behaviors, eating behaviors, and change in BM, partial correlation analysis was conducted controlling for program type. The assumptions of multiple regression were checked before conducting hierarchical multiple regression analysis, controlling for program type, to assess whether WL was significantly predicted by early (week three) and early-late change (difference between week three and week 12) in free-living PA, SB, energy intake, macronutrient intake, or energy density. A linear regression analysis was conducted to explore whether self-reported program compliance over the 14 weeks intervention significantly predicted weight change. The difference in baseline sample characteristics between the CWL (≥5% reduction in BM) and NWL (≤3% reduction in BM) were assessed using univariate ANCOVA controlling for program type. To assess group differences in changes in BM or BMI, 3 (week: zero, two, and 14) x 2 (groups: CWL and NWL) mixed ANCOVAs controlling for program type were performed. Univariate ANCOVAs were then conducted to explore the significant interaction effects. To compare changes in free-living PA, SB, energy intake and macronutrient intake data between groups, 2 (week: two and 14) x 2 (group: CWL and NWL) mixed ANCOVAs were performed controlling for program type. The effect of the covariate was only reported where significant. Greenhouse-Geisser probability levels were used to adjust for sphericity, only if appropriate. All main effects and interaction effects were examined with Bonferroni *post-hoc* tests. Partial eta squared (η^2^) is reported for effective sizes and interpreted as follows: small, 0.01; medium, 0.06; large, 0.14 (40).

Statistical analysis was performed using IBM SPSS for Windows (Chicago, IL, USA, Version 24) and significance was set at *p* < 0.05 except for tests with multiple comparisons, in which case, a more conservative *p*-value was used to account for multiple comparisons [0.05 divided by the number of comparisons (three comparisons = *p* < 0.017)]. The analysis plan was registered prior to conducting the data analysis using the secondary data preregistration template on the Open Science Framework (OSF; https://osf.io/fptwn).

## Results

### Study Population and Attrition Rates

In total, 613 individuals (291 commercial program) responded to the various recruitment methods. Of those, 517 were excluded for various reasons and 96 (49 commercial programs) were recruited to the study. A further 16 either withdrew from the study or were excluded resulting in a final sample of 80 (37 commercial programs). Of the 80 participants who completed the study, 41 lost ≥5% of their initial BM. The details of the recruitment process and reasons for exclusion and attrition from the study have been reported previously (29). In this primary article, an additional two participants were excluded (*n* = 78) as there were delays with some elements of data collection, but those data are not being reported in this article, so the participants were retained. The participants were aged 42.0 ± 12.4 years with a BMI of 34.08 ± 3.62 kg/m^2^. Average self-reported compliance (How well you have managed to stick with the weight control program?) across the 14-week program for those who completed the study was 48.0 ± 20.9 mm. During the 3rd week, the average SWA wear time was 1,412.1 ± 21.3 min/day (98.1%) and during the 12th week, the average SWA wear time was 1,416.5 ± 12.4 min/day (98.4%). The data from the SWA were missing for 15 participants because they either did not want to wear the SWA (*n* = 1), they did not comply with the wear procedure (*n* = 11), or the data file was lost/corrupted (*n* = 3). In addition, food diary data were missing for three participants because they did not complete the food diary.

### Pooled Data

#### Changes in Body Mass, Body Mass Index, and Body Composition

When whole sample data were analyzed, there was a significant reduction in BM at each time point [η*p*^2^ = 0.284; *p* < 0.001]: baseline [91.46 ± 12.61 kg (88.65, 94.27 kg)], week two [89.11 ± 12.61 kg (86.31, 91.91 kg)], and week 14 [87.05 ± 13.01 kg (84.16, 89.95kg)], *post-hoc* results between baseline and week 2, baseline and week 14, and week 2 and week 14 were all *p* < 0.001. Therefore, there was a significant reduction in BMI at each time point [η*p*^2^ = 0.274; *p* < 0.001]: baseline [34.08 ± 3.64 kg/m^2^ (33.27, 34.89 kg/m^2^)], week two [33.20 ± 3.60 kg/m^2^ (32.40, 34.00 kg/m^2^)] and week 14 [32.44 ± 3.88 kg/m^2^ (31.57, 33.30 kg/m^2^)], *post-hoc results* between baseline and week two, baseline and week 14, and week two and week 14 were all *p* < 0.001. There was a significant interaction between week and program type for BM [*p* = 0.001] and BMI [*p* = 0.001].

There was a significant reduction in FM [η*p*^2^ = 0.22; *p* < *0.001*] from week two [41.46 ± 9.97 kg (39.03, 43.90 kg)] to week 14 [39.55 ± 10.30 kg (37.04, 42.07 kg)] and a significant interaction between week and program type for FM [*p* = 0.009]. There was no significant change in FFM [η*p*^2^ = 0.01; *p* = 0.48] from week two [48.01 ± 5.97 kg (46.56, 49.47 kg)] to week 14 [48.03 ± 5.99 kg (46.57, 49.49 kg)].

#### Changes in Free-Living Movement and Eating Behaviors

There was a significant increase in percentage fat intake [η*p*^2^ = 0.05; *p* = 0.05] from week three [32.76 ± 4.47% (31.75, 33.77%)] to week 12 [34.19 ± 5.77% (32.88, 35.50%)]. There were no other significant changes in any of the other free-living movement or eating behaviors [largest η*p*^2^ = 0.04; smallest *p* = 0.10]. There was a significant interaction between week and program type for light PA [*p* = 0.03]. LOCF analyses did not differ (data not shown).

#### Behavioral Predictors of Body Mass Change

Partial correlations showed that higher vigorous PA [*p* = 0.01], higher percentage CHO intake [*p* = 0.03], lower total EI [*p* = 0.01], and lower energy-dense [*p* < 0.001] foods consumed early in the intervention (week three) were associated with a greater reduction in BM. An increase in vigorous PA [*p* < *0.001*] and a decrease in SB [*p* = 0.03] from early (week three) to late (week 12) in the intervention were also associated with greater WL. All other movements and eating behavior variables were not significantly associated with BM changes (see Table 1).

**Table 1:** Association between early (week three) and early-late change (week three to week 12) movement and eating behaviors and change in body mass (BM) between baseline and week 14.

	**Total EE (kcal/d)**	**Light PA (min/d)**	**Moderate PA (min/d)**	**Vigorous PA (min/d)**	**SB (min/d)**	**Total EI (kcal/d)**	**Carbohydrate (%)**	**Fat (%)**	**Protein (%)**	**Energy density (kcal/g)**
	**Early (week 3)**									
ΔBM (%)	0.06	−0.19	−0.10	−0.32**	0.10	0.28**	−0.25*	0.20	−0.08	0.37***
	**Early-late change (Δ)**									
ΔBM (%)^1^	0.12	−0.11	−0.18	−0.38**	0.27*	−0.05	0.04	0.15	−0.08	0.08
ΔBM (%)^2^	0.13	−0.08	−0.19	−0.37**	0.25*	−0.04	0.01	0.10	−0.12	0.80

*Data are partial correlations controlling for program type*.

Asterisks indicate that the differences are significant (***p < 0.001; **p < 0.01; *p < 0.05).

*BM (%) is a percentage change from baseline to week 14*.

1 *Completer sample*.

2 *LOCF sample*.

*Early (week 3) movement behavior correlations n = 77; early (week 3) eating behavior correlations n = 80; early-late change in movement behavior correlations for completer sample n = 65; early-late change in eating behavior correlations n = 77. Bold values indicate the correlation is significant*.

Hierarchical linear regression analyses were conducted to evaluate the prediction of percentage BM change from movement and eating behaviors. Program type was controlled for and entered as a covariate in the first step of each regression model (forced entry). The movement and eating behavior variables [total energy expenditure (EE), light, moderate, and vigorous PA, SB, total EI, macronutrient composition, and energy density] were entered in step two using the stepwise method. Two separate hierarchical multiple regressions were conducted to determine the unique contributions of early (model one) and early-late change (model two) in movement and eating behaviors to percentage BM change.

Model one revealed that the energy density of foods consumed and vigorous PA early in the intervention (week three) significantly predicted 29.0% of the variance in percentage BM change (as shown in Table 2). The results from model two demonstrated that early-late change in light PA, moderate PA, vigorous PA, total EE, and energy density of foods consumed significantly predicted 73% of the variance in percentage BM change. These hierarchical linear regression analyses demonstrate that higher week three vigorous PA and an increase in light, moderate, and vigorous PA were associated with greater WL. Conversely, higher week three energy density and an increase in total EE and energy density were associated with less WL. The LOCF analyses results did not differ (as shown in Supplementary Table 1).

**Table 2:** Hierarchical linear regression analyses predicting change in percentage BM between baseline and week 14 from week three movements and eating behaviors and from early-late change in movement and eating behaviors.

**Model**	**Variables**	**B (95% CI)**	**SE B**	**β**	***p***	**F**	**R^**2**^**	**ΔR^**2**^**
**Predictor variables: Early (week 3) movement and eating behaviors**								
1	–	–	–	–	–	9.95	0.29	0.09
	Constant	−11.62 (−16.07, −7.17)	2.23	–	<0.001	–	–	–
	Program type	−0.43 (−2.44, 1.59)	1.01	−0.05	= 0.67	–	–	–
	Energy density (kcal/g)	5.69 (2.79, 8.58)	1.45	0.45	<0.001	–	–	–
	Vigorous PA (min/d)	−0.38 (−0.63,−0.13)	0.12	−0.30	= 0.003	–	–	–
**Predictor variable: Early-late change (Δ) in movement and eating behaviors**								
2	–	–	–	–	–	25.03	0.73	0.03
	Constant	−3.13 (−4.15,−2.11)	0.51	–	<0.001	–	–	–
	Program type	−0.85 (−2.17, 0.48)	0.66	−0.09	= 0.21	–	–	–
	Δ Vigorous PA (min/d)	−0.47 (−0.62,−0.32)	0.07	−0.49	<0.001	–	–	–
	Δ Total EE (kcal/d)	0.03 (0.03, 0.04)	0.01	1.84	<0.001	–	–	–
	Moderate PA (min/d)	−0.13 (−0.17,−0.10)	0.02	−1.17	<0.001	–	–	–
	Δ Light PA (min/d)	−0.05 (−0.07,−0.04)	0.01	−0.81	<0.001	–	–	–
	Energy density (kcal/g)	2.83 (0.59, 5.08)	1.12	0.18	= 0.01	–	–	–

*Unstandardized beta (B), SE for the unstandardized beta (SE B), standardized beta (β), N = 63*.

*Model two was conducted on the completer sample*.

### Individual Variability in Body Mass Change

#### Analysis of Clinically Significant Weight Losers and Non-Clinically Significant Weight Losers Sample Characteristics

There was considerable individual variability in BM change among the participants ranging from −18.02 to +3.20 kg (−17.68 to +3.50%) with six (8.1%) participants gaining weight. Self-reported program compliance over the 14-week intervention significantly predicted weight change [F(1, 64) = 33.19, *p* < 0.001, *R*^2^ = 0.34]. CWL reported significantly greater compliance with the program compared with NWL [CWL: 58.6 ± 17.4 mm (52.9, 64.3 mm); NWL: 35.3 ± 17.3 mm (28.7, 42.0 mm), *t*(64) = 5.38, *p* < 0.001].

#### Between Group Comparison of Changes in Body Mass

There were no differences between CWL and NWL early in the intervention for BM (η*p*^2^ = 0.005; *p* = 0.55), BMI (η*p*^2^ = 0.027; *p* = 0.16), FM (η*p*^2^ = 0.019; *p* = 0.29), or FFM (η*p*^2^ = 0.001; *p* = 0.83). There was a main effect of week for BM (η*p*^2^ = 0.269; *p* < 0.001) and *post-hoc* tests showed that BM differed significantly between all three time points [*post-hoc* results all *p* < 0.001] (as shown in Table 3). There was also a week x group interaction on BM [ηp^2^ = 0.526; p < 0.001] that revealed CWL lost significantly more weight between all three time-points compared with NWL: baseline and week two [CWL: −3.09 ± 1.10 kg (−3.44, −2.75 kg); NWL: −1.52 ± 1.10 kg (−1.90, −1.13 kg), η*p*^2^ = 0.338; *p* < 0.001]; baseline and week 14 [CWL: −7.21 ± 2.54 kg (−8.00, −6.42 kg); NWL: −1.12 ± 2.55 kg (−2.01, −0.23 kg), η*p*^2^ = 0.588; *p* < 0.001]; and weeks two and 14 [CWL: −4.12 ± 2.39 kg (−4.86, −3.37 kg); NWL: 0.40 ± 2.40 kg (−0.44, −1.23 kg), η*p*^2^ = 0.469; *p* < 0.001]. There was a significant interaction between week and program type for BM [*p* = 0.02]. Refer to Section 2 of the Supplementary Materials for between group comparison of changes in BMI and body composition.

**Table 3:** Change in body mass (BM) and body mass index (BMI) between baseline, week 2 and week 14.

	**Group**	***n***	**Baseline**	**Week 2 (early)**	**Week 14 (late)**	**Change (Δ)**
BM (kg)	CWL	41	91.06 ± 13.15 (86.97, 95.15)	87.97 ± 13.03 (83.91, 92.02) ^**a**^	83.05 ± 12.99 (79.80, 87.90) ^b^	−7.21 ± 2.54 (−8.00,−6.42)***
	NWL	33	92.94 ± 13.19 (88.36, 97.52)	91.43 ± 13.07 (86.89, 95.97) ^**a**^	91.82 ± 13.05 (87.29, 96.35) ^b^	−1.12 ± 2.55 (−2.01,−0.23)
BMI (kg/m^2^)	CWL	41	33.68 ± 3.75 (32.51, 34.86)	32.52 ± 3.66 (31.38, 33.66) ^c^	31.00 ± 3.69 (29.85, 32.15) ^**d**^	−2.68 ± 0.97 (−2.98, −2.38)***
	NWL	33	34.95 ± 3.77 (33.64, 36.35)	34.37 ± 3.67 (33.10, 35.65) ^c^	34.52 ± 3.71 (33.23, 35.80) ^**d**^	−0.43 ± 0.98 (−0.77, −0.09)

*Data are adjusted M ± SD (95% CI)*.

*Data are estimated marginal means adjusted for program type*.

*The change represents the difference between baseline and week 14*.

Asterisks indicate the differences are significant (^***^ p < 0.001) and when necessary superscript letters are used to indicate differences between the groups, i.e., the same letter is used for any pair when there is a significant difference observed (if bold p < 0.01, otherwise p < 0.05).

#### Between Group Comparison of Change in Free-Living Movement and Eating Behaviors

Early in the intervention (week three), the energy density of the foods consumed by CWL was significantly lower than the energy density of the foods consumed by NWL [η*p*^2^ = 0.071; *p* = 0.03] (as shown in Table 4). However, there were no statistically significant differences between groups in early (week three) measures of total EE, light PA, moderate PA, vigorous PA, SB, total EI, percentage carbohydrate intake, percentage fat intake, and percentage protein intake [largest η*p*^2^ = 0.071; smallest *p* = 0.08].

**Table 4:** Change in energy expenditure (EE), free-living physical activity [from light to vigorous physical activity (PA)], sedentary behavior (SB), energy intake, and macronutrient composition between week 3 and week 12.

	**Group**	***n***	**Week 3 (early)**	**Week 12 (late)**	**Early-late change (Δ)**
**Total EE (kcal/d)**	CWL	37	2606.36 ± 356.07 (2489.19, 2723.54)	2552.10 ± 370.84 (2430.06, 2674.14)	−54.27 ± 251.18 (−136.92, 28.39)
	NWL	24	2530.44 ± 357.23 (2384.47, 2676.41)	2487.28 ± 372.06 (2335.26, 2639.31)	−42.16 ± 252.00 (−146.13, 59.81)
**Light PA (min/d)**	CWL	37	201.64 ± 73.88 (177.33, 225.95)	191.29 ± 73.88 (165.79, 216.80)	−10.35 ± 66.17 (−32.13, 11.43)
	NWL	24	178.96 ± 74.13 (148.67, 209.25)	154.05 ± 77.75 (122.28, 185.81)	−24.91 ± 66.40 (−52.04, 2.22)
**Moderate PA (min/d)**	CWL	37	75.71 ± 48.11 (59.88, 91.55)	89.18 ± 55.79 (70.82, 107.54)	13.46 ± 39.97 (0.31, 26.62)
	NWL	24	67.31 ± 48.27 (47.59, 87.03)	65.40 ± 55.97 (42.53, 88.27)	−1.91 ± 40.11 (−18.29, 14.48)
**Vigorous PA (min/d)** ^†^	CWL	37	2.22 ± 3.87 (0.95, 3.94)	4.23 ± 5.07 (2.56, 5.90)	2.01 ± 4.64 (0.49, 3.54)*
	NWL	24	1.39 ± 3.88 (-0.19, 2.98)	0.80 ± 5.09 (-1.28, 2.88)	−0.60 ± 4.65 (−2.50, 1.31)
**SB (min/d)**	CWL	37	717.77 ± 99.48 (685.03, 750.51)	706.99 ± 109.61 (670.93, 743.06)	−10.78 ± 100.30 (−43.78, 22.23)
	NWL	24	746.45 ± 99.81 (705.67, 787.23)	768.45 ± 109.97 (723.52, 813.38)	22.00 ± 100.63 (−19.12, 63.12)
**Total EI (kcal/d)**	CWL	40	1538.27 ± 448.30 (1396.83, 1679.72)	1536.40 ± 435.97 (1398.85, 1673.95)	−1.87 ± 395.53 (−126.63, 122.90)
	NWL	31	1702.77 ± 449.69 (1111.40, 1863.94)	1595.28 ± 437.33 (437.33, 1752.02)	−107.49 ± 396.72 (−249.67, 34.21)
**Carbohydrate intake (%)**	CWL	40	46.04 ± 6.30 (44.06, 48.03)	44.71 ± 8.17 (42.13, 47.28)	−1.34 ± 6.48 (−3.38, 0.71)
	NWL	31	43.37 ± 6.32 (41.10, 45.63)	41.91 ± 8.20 (38.98, 44.85)	−1.45 ± 6.50 (−3.78, 0.88)
**Carbohydrate intake (kcal/d)**	CWL	40	708.22 ± 96.91 (677.73, 738.80)	686.92 ± 125.52 (647.29, 726.41)	−21.30 ± 175.08 (−79.88, 107.44)
	NWL	31	738.49 ± 107.62 (699.84, 776.97)	668.58 ± 130.81 (621.84, 715.48)	−69.91 ±175.70 (−110.15, 15.82)
**Fat intake (%)**	CWL	40	32.01 ± 4.56 (30.58, 33.45)	32.84 ± 5.88 (30.99, 34.70)	0.83 ± 5.70 (−0.97, 2.63)
	NWL	31	33.44 ± 4.57 (31.80, 35.08)	35.58 ± 5.90 (33.47, 37.69)	2.14 ± 5.72 (0.09, 4.19)
**Fat intake (kcal/d)**	CWL	40	492.40 ± 70.14 (470.38, 514.53)	504.55 ± 90.34 (476.13, 533.13)	12.15 ± 182.72 (−41.81, 73.52)
	NWL	31	569.41 ± 77.82 (541.48, 597.33)	567.60 ± 94.12 (533.94, 601.26)	−1.81 ± 183.35 (−80.65, 50.81)
**Protein intake (%)**	CWL	40	19.06 ± 3.00 (18.11, 20.01)	18.95 ± 3.61 (17.81, 20.09)	−0.12 ± 4.11 (−1.41, 1.18)
	NWL	31	19.64 ± 3.02 (18.56, 20.72)	19.99 ± 3.62 (18.70, 21.30)	0.36 ± 4.13 (−1.12, 1.84)
**Protein intake (kcal/d)**	CWL	40	293.19 ± 46.15 (278.57, 307.79)	291.15 ± 55.46 (273.63, 308.66)	−2.04 ± 71.16 (−27.12, 17.78)
	NWL	31	334.42 ± 51.42 (316.03, 352.81)	318.90 ± 57.75 (298.32, 339.79)	−15.52 ± 71.40 (−45.90, 5.30)
**Energy density (kcal/g)** ^†^	CWL	40	1.24 ± 0.27 (1.15, 1.32) ^a^	1.36 ± 0.30 (1.26, 1.45)	0.12 ± 0.31 (0.02, 0.22)
	NWL	31	1.39 ± 0.31 (1.29, 1.48) ^a^	1.47 ± 0.30 (1.36, 1.58)	0.08 ± 0.31 (−0.03, 0.19)

*Data are adjusted M ± SD (95% CI)*.

*Data from the SenseWear Armband were missing for 15 participants because they either did not want to wear the SWA, they did not comply with the wear procedure or the data file was lost/corrupted. Food diary data were missing for three participants because they did not complete the food diary*.

Asterisks indicate early-late change is significant (^*^ p < 0.05).

† *indicates the main effect of group is significant; and when necessary superscript letters are used to indicate differences between the groups, i.e., the same letter is used for any pair when there is a significant difference observed (if bold p < 0.01, otherwise p < 0.05)*.

For early-late change in the movement and eating behaviors, CWL showed a small but significant increase in vigorous PA, whereas NWL showed a slight decrease [significant week x group interaction, η*p*^2^ = 0.072; *p* = 0.04, as shown in Table 4]. The main effect of group for vigorous PA was significant; CWL [3.23 ± 3.87 min/day (1.95, 4.50 min/day)] performed more vigorous PA on average than NWL [1.10 ± 3.88 min/day (−0.49, 2.68 min/day), η*p*^2^ = 0.070; *p* = 0.04]. On average, the energy density of foods consumed was lower in CWL [1.30 ± 0.24 kcal/g (1.22, 1.37 kcal/g)] compared with NWL [1.43 ± 0.24 kcal/g (1.34, 1.52 kcal/g), η*p*^2^ = 0.070; *p* = 0.03]. There were no other main effects of week or group and no other week x group interactions for movement behaviors or eating behaviors.

The LOCF sample analyses (as shown in Supplementary Table 2) were much the same with the addition of a significant main effect of group for percentage fat intake; CWL [32.34 ± 4.49% (30.95, 33.74%)] consumed less fat on average than NWL [34.71 ± 4.51% (33.14, 36.27%), ηp^2^ = 0.064; *p* = 0.03].

## Discussion

The results of this study demonstrate that early and early-late change in both free-living movement and eating behaviors are associated with weight loss following a weight loss program focused primarily on diet. When whole sample data were analyzed, higher vigorous PA, higher percentage CHO intake, lower total EI, and lower energy density foods consumed early in the intervention (week three) were associated with a greater reduction in BM. In addition, an increase in vigorous PA and a decrease in SB from early (week three) to late (week 12) in the intervention were also associated with greater WL. Free-living movement and eating behaviors were also predictive of BM change. Consuming lower energy-dense foods and engaging in greater vigorous PA early in the intervention significantly predicted greater weight loss. As did early-late increases in light PA, moderate PA, vigorous PA, decreases in total EE and energy density of foods consumed. When participants were categorized based on their WL response, those who experienced the most successful weight loss (CWL) consumed lower energy-dense foods early in the intervention and on average, they showed a significant early-late increase in vigorous PA and performed more vigorous PA on average. Collectively, these findings demonstrate that specific behaviors that contribute to greater EE [e.g., vigorous PA (31)] and lower EI [e.g., less energy dense foods (29)] were related to better WL outcomes.

The current findings showing that the amount of PA is related to WL align with previous research which found that higher PA prior to engaging in an aerobic exercise intervention (41) and greater PA levels during a WL program were associated with greater WL (42, 43). Research examining whether energy balance behaviors (i.e., movement and eating behaviors), early in the intervention, and the change in those behaviors during an intervention, are predictive of WL success following a WL program primarily focused on diet is lacking. The present study confirms the findings from Vaanholt et al. (44) in women with overweight or obesity; free-living PA (particularly vigorous PA) early in the intervention and early-late change significantly predicted weight loss. Interestingly, early-late change in behavior predicted more of the variability is BM change (73%) than the model using early intervention data (29%). This suggests that change in behavior during a WL program is a more important determinant of weight loss success. To optimize WL, strategies to monitor energy balance behaviors during a weight loss intervention could identify individuals who may benefit from additional support. Light PA and moderate PA early in the intervention were not significant predictors of WL, but an early-late change in those behaviors was predictive of WL. Encouraging participants to replace SB with light PA and moderate PA early in the intervention could be one potential strategy to promote WL (45). Particularly, since a decrease in SB during the intervention was associated with greater WL. Interestingly, an early-late increase in total EE, which is heavily influenced by RMR which, in turn, is dependent on BM, was predictive of poorer WL outcomes in the current study. This finding appears counter-intuitive at first, but a probable explanation is that the total EE algorithm within the SWA software was influenced by the individuals who gained weight. BM is part of the SWA algorithm for estimating EE and an increase in BM (with an associated increase in resting metabolic rate) would result in an increase in total EE with no change in PA. Another possible explanation is that increased EE was driving an increase in EI, as proposed previously (46, 47), resulting in poorer WL.

Individuals who experienced the most WL (CWL) had significant differences in PA behavior profiles compared with those who experienced less WL (NWL). The CWL group significantly increased their vigorous PA whereas the NWL group showed a slight reduction. Furthermore, those who lost more weight performed more vigorous PA on average. Previous research has highlighted the role of vigorous PA in weight management (48). In this study, the increase in vigorous PA in the CWL group was small (~2 min/day) and would have minimal impact on energy expenditure. However, the increase in vigorous PA could be large enough to positively impact other health outcomes. A recent review concluded low-volume high-intensity interval training protocols, with a similar amount of vigorous PA to the increase observed in the CWL group, has no effect on body fat or BM, a tendency to improve FFM (although not statistically significant) and favorable effects on various health outcomes, such as cardiorespiratory fitness (49). The observed increase in vigorous PA may reflect concerted efforts to increase purposeful structured exercise rather than incidental PA and potentially resulted in greater compliance with the WL diet as has been previously reported (50). Indeed, the CWL group self-reported significantly higher compliance with the program.

The consumption of lower energy-dense foods and lower total EI early in the intervention were associated with greater WL. Furthermore, consumption of lower energy-dense foods early in the intervention and an early-late decrease in energy-dense foods was predictive of successful WL. These findings are in line with previous research concluding that the consumption of a diet lower in energy-dense foods may be an effective strategy for managing body weight (51). Those who achieved clinically significant WL also exhibited different eating behavior to those who did not. Early in the intervention and on average, the CWL group consumed lower energy-dense foods compared with the NWL group. This supports previous findings demonstrating consumption of a low energy-dense diet leads to weight loss through improved appetite control and reduced EI (29). There was also a trend toward the main effect of week and group for percentage fat intake such that percentage fat intake was higher in the NWL group and there was an early-late increase in the percentage fat intake (but not absolute fat intake). The increase in fat intake during the intervention could indicate a decrease in adherence to the WL diet (52), a weakening in restraint/increase in disinhibition (21) or a compensatory response to prevent further WL (53). The types of fat participants consumed were not measured, therefore it is not possible to comment on the quality of fats consumed by participants in this study (54).

This study supports previous research reporting that early WL is an important marker of program success and long-term WL outcomes (18). In the current study, those who lost ≥5% of their initial BM (CWL) showed a statistically significant reduction in BM at each time point (including baseline to week two), whereas those who lost ≤ 3% of their initial BM (NWL) did not. This provides further support for the use of early non-response to WL programs as a marker for identifying individuals who could benefit from additional support. Unick et al. (18) recommend the use of adaptive or stepped care interventions to provide an individualized program for those in need of additional help. Additional research is needed to explore the optimal time point to intervene, the threshold for identifying those in need of additional support, and the type of intervention that is most effective for boosting WL in early non-responders. In the present study, strategies early in the intervention to improve compliance, promote PA (particularly vigorous PA), and reduce the consumption of energy dense foods may have promoted greater WL in those with poorer WL outcomes.

Subtracting the EI data from the estimates of EE suggests that the CWL were in an energy deficit of ~1068–1016 kcal/day, while the NWL were in an energy deficit of ~827–892 kcal/day. Assuming EI and EE remained the same, that would give an energy deficit of ~100,000 kcal for the CWL and ~85,000 kcal for the NWL over the study. Assuming 1 kg of BM (70:30 fat/lean tissue) is equivalent to 7,000 kcal (55), based on the observed BM changes, the predicted energy deficit would be 50,470 kcal and 7,840 kcal for the CWL and NWL, respectively. These calculations highlight the well-documented issue of underreporting inherent with self-reported dietary intake, particularly in people with obesity (56, 57). Interestingly, those who lost less weight underreported their EI to a greater extent (~780 kcal/day) than those who lost more weight (~55 kcal/day). It is acknowledged that the calculations are not accurate (58); however, the 7,000 kcal rule provides an indication of the energy deficit required to produce the observed weight losses vs. the energy deficit calculated from the EI and EE data. These considerations highlight the potential problems with self-report dietary variables as the potential predictors of weight outcomes. If it is assumed that the energy deficits estimated from the PA assessments in combinations with body weight are likely to be more accurate than those using self-report dietary intakes, this would mean that EI was underreported by approximately 30% in the NWL group and by about 2–3% in the CWL group. This has implications for the combined predictor and the group comparisons presented above. Interestingly, it also implies that those who are more successful at WL are able to more accurately assess their true EI over time. These considerations are part of an on-going analysis of dietary misreporting as a predictor of successful WL.

There are several limitations inherent in this study that should be acknowledged. First, due to the restrictions around participant recruitment, PA and EI were not fully captured at baseline and some adaptations may already have occurred in the first 2 weeks of the intervention. Changes identified between early and late measurement periods may have reflected a regression back to baseline, for example, the increase in percentage fat intake, limiting the interpretability of these findings. However, early and early-late changes in PA and EI were still predictive of WL success. Second, while the data suggests adherence to the WL program may play a role in WL outcomes, there are limitations with this measure. There is no accepted or feasible method of measuring adherence to WL programs (18). In this study, adherence was self-reported and may have been confounded by participants knowing whether they lost weight each week. PA promotion was a component of the WL programs and accelerometer-based measures of free-living PA were positively correlated with adherence (data not presented) supporting the validity of the adherence measure. Third, WL was induced using two different WL programs (commercial and self-led), but because there were no specific research questions pertaining to program type, WL data were analyzed with both WL program groups combined and program type was controlled for in all the analyses. However, the two WL programs were inherently different. It is therefore difficult to disentangle whether reduced energy density was a predictor of WL success, given the group that was placed on the low energy dense diet lost the most weight (29), or whether it was due to the face-to-face support that group received, or a combination of both. Fourth, although it is an accepted and widely used energy density calculation, the Wrieden method did not account for the calories consumed in drinks (other than milk). Fifth, the stage of the menstrual cycle was not controlled for and therefore a confounding effect on energy intake cannot be ruled out (59). Finally, to overcome the attrition in this study, which is common in WL interventions (60), missing data was imputed using the last observation carried forwards method. This method has previously been implemented in WL trials (61) and the limitations of this approach have previously been acknowledged (62). A strength of the current study is the measurement of PA (for ~23 h/day) and EI under free-living conditions over a complete week during the early and late stages of the intervention with strong and sensitive measurement methods.

### Conclusion

Early and early-late change in free-living movement and eating behaviors during a 14-week WL program are predictors of WL. These findings demonstrate that specific behaviors that contribute to greater EE (e.g., vigorous PA) and lower EI (e.g., less energy-dense foods) are related to more successful WL outcomes. Interventions targeting these behaviors may increase the effectiveness of WL programs. Additional research is needed to explore the threshold for identifying those in need of additional support, the optimal time point to intervene, and the type of intervention that is most effective for boosting WL in those in need of additional support.

## Data Availability Statement

The data analyzed in this study is subject to the following licenses/restrictions: The current datasets are available from the corresponding author on reasonable request. Requests to access these datasets should be directed to Anna Myers, a.myers@shu.ac.uk.

## Ethics Statement

This study involved human participants and was reviewed and approved by The School of Psychology Research Ethics Committee at the University of Leeds (14-0090). The patients/participants provided their written informed consent to participate in this study.

## Author Contributions

AM, NB, CG, JB, and GF designed the research. NB, DC, and FC conducted the trial. AM processed the physical activity data, performed statistical analyses, and wrote the manuscript. All authors read and approved the final manuscript.

## Funding

The trial was funded by Slimming World UK. Slimming World UK supported recruitment. The funder had no role in the analysis or writing of this article.

## Conflict of Interest

The authors declare that the research was conducted in the absence of any commercial or financial relationships that could be construed as a potential conflict of interest.

## Publisher's Note

All claims expressed in this article are solely those of the authors and do not necessarily represent those of their affiliated organizations, or those of the publisher, the editors and the reviewers. Any product that may be evaluated in this article, or claim that may be made by its manufacturer, is not guaranteed or endorsed by the publisher.
