# Supplementary material for: Free-Living Energy Balance Behaviors Are Associated With Greater Weight Loss During a Weight Loss Program
Source: Front Nutr. 2021 Sep 14;8:688295. doi: 10.3389/fnut.2021.688295 (PMC8478016; doi:10.3389/fnut.2021.688295)
Supplement: Supplementary file 1 [file Data_Sheet_1.docx]

Supplementary Material

# Supplementary Data

Supplementary table 1. LOCF hierarchical linear regression analyses predicting change in % BM between baseline and week 14 from early-late change in movement and eating behaviours.

| **Model** | **Variables** | **B (95% CI)** | **SE B** | **β** | ***p*** | **F** | **R^2^** | **△R^2^** |
| --- | --- | --- | --- | --- | --- | --- | --- | --- |
| **Predictor variable: Early-late change (△) in movement and eating behaviours** | | | | | | | | |
| **2** | - | - | - | - | - | 28.30 | .71 | .02 |
|  | Constant | -2.82 (-3.67, -1.97) | 0.43 | - | < .001 | - | - | - |
|  | Programme type | -1.13 (-2.30, 0.05) | 0.59 | -0.13 | = .060 | - | - | - |
|  | △ Vigorous PA (min/d) | -0.48 (-0.62, -0.33) | 0.07 | -0.46 | < .001 | - | - | - |
|  | △ Total EE (kcal/d) | 0.03 (0.03, 0.04) | 0.01 | 1.74 | < .001 | - | - | - |
|  | △ Moderate PA (min/d) | -0.14 (-0.17, -0.10) | 0.02 | -1.12 | < .001 | - | - | - |
|  | △ Light PA (min/d) | -0.05 (-0.07, -0.04) | 0.01 | -0.75 | < .001 | - | - | - |
|  | Energy density (kcal/g) | 2.18 (0.21, 4.16) | 0.99 | 0.14 | = .031 | - | - | - |
| Unstandardised beta (B), standard error for the unstandardised beta (SE B), standardised beta (β), N = 77.  Model two was conducted using the LOCF sample. | | | | | | | | |

Supplementary table 2. LOCF analyses exploring change in energy expenditure (EE), free-living physical activity (from light to vigorous physical activity [PA]), sedentary behaviour (SB), energy intake and macronutrient composition between week 3 and 12. Data are adjusted *M* ± *SD* (95% confidence intervals).

|  | **Group** | **n** | **Week 3 (early)** | **Week 12 (late)** | **Early-late change (△)** |
| --- | --- | --- | --- | --- | --- |
| **Total EE (kcal/d)** | CWL | 40 | 2605.257 ± 346.69 (2495.90, 2714.61) | 2553.877 ± 362.42 (2439.56, 2668.20) | -51.38 ± 233.36 (-124.98, 22.23) |
|  | NWL | 32 | 2572.882 ± 347.74 (2450.25, 2695.52) | 2541.994 ± 64.26 (2413.80, 2670.19) | -30.89 ± 234.06 (-113.43, 51.66) |
| **Light PA (min/d)** | CWL | 40 | 199.19 ± 72.26 (176.40, 221.99) | 189.15 ± 76.74 (164.94, 213.35) | -10.04 ± 62.28 (-29.69, 9.60) |
|  | NWL | 32 | 185.52 ± 72.49 (159.96, 211.09) | 167.43 ± 76.97 (140.28, 194.58) | -18.09 ± 62.46 (-40.12, 3.93) |
| **Moderate PA (min/d)** | CWL | 40 | 74.64 ± 47.08 (59.79, 89.48) | 86.80 ± 53.75 (69.85, 103.76) | 12.17 ± 37.03 (0.486, 23.85) |
|  | NWL | 32 | 65.06 ± 47.22 (48.41, 81.72) | 63.99 ± 53.91 (44.98, 83.00) | -1.07 ± 37.14 (-14.17, 12.03) |
| **Vigorous PA (min/d)** ✝ | CWL | 40 | 2.10 ± 3.65 (0.95, 3.25) | 3.99 ± 4.81 (2.47, 5.51) | 1.89 ± 4.31 (0.53, 3.25)* |
|  | NWL | 32 | 1.20 ± 3.66 (-0.09, 2.49) | 0.72 ± 4.83 (-.98, 2.42) | -0.48 ± 4.32 (-2.00, 1.05) |
| **SB (min/d)** | CWL | 40 | 719.58 ± 95.69 (689.40, 749.76) | 710.29 ± 105.43 (677.03, 743.55) | -9.29 ± 93.28 (-38.71, 20.13) |
|  | NWL | 32 | 740.60 ± 95.98 (706.76, 774.45) | 756.26 ± 105.75 (718.96, 793.55) | 15.65 ± 93.56 (-17.34, 48.65) |
| **Total EI (kcal/d)** | CWL | 41 | 1558.50 ± 464.34 (1413.91, 1703.10) | 1558.96 ± 455.11 (1417.24, 1700.69) | 0.46 ± 388.72 (-120.59, 121.51) |
|  | NWL | 33 | 1710.02 ± 465.97 (1548.28, 1871.75) | 1606.19 ± 456.71 (1447.66, 1764.72) | -103.83 ± 390.10 (-239.23, 31.58) |
| **Carbohydrate intake (%)** | CWL | 41 | 46.15 ± 6.29 (44.19, 48.10) | 44.85 ± 8.13 (42.32, 47.39) | -5.91 ± 45.65 (-20.13, 8.30) |
|  | NWL | 33 | 43.38 ± 6.31 (41.19, 45.57) | 41.99 ± 8.16 (39.17, 44.83) | -12.43 ± 45.81 (-28.33, 3.47) |
| **Fat intake (%)** ✝ | CWL | 41 | 31.96 ± 4.62 (30.52, 33.40) | 32.73 ± 5.89 (30.90, 34.57) | 1.88 ± 19.83 (-4.30, 8.05) |
|  | NWL | 33 | 33.68 ± 4.64 (32.07, 35.29) | 35.74 ± 5.92 (33.68, 37.79) | -1.75 ± 19.90 (-8.66, 5.16) |
| **Protein intake (%)** | CWL | 41 | 19.08 ± 3.00 (18.15, 20.02) | 18.96 ± 3.59 (17.84, 20.08) | -0.83 ± 17.63 (-6.32, 4.66) |
|  | NWL | 33 | 19.53 ± 3.02 (18.48, 20.57) | 19.88 ± 3.60 (18.63, 21.13) | 0.36 ± 4.13 (-1.12, 1.84) |
| **Energy density (kcal/g)** ✝ | CWL | 41 | 1.24 ± 0.29 (1.15, 1.33) ^a^ | 1.36 ± 0.32 (1.25, 1.45) | 0.11 ± 0.30 (0.02, 0.21) |
|  | NWL | 33 | 1.42 ± 0.30 (1.31, 1.52) ^a^ | 1.49 ± 0.32 (1.38, 1.60) | 0.08 ± 0.30 (-0.03, 0.18) |
| Data from the SenseWear Armband were missing for 2 participants because they did not want to wear the SWA or they did not comply with the wear procedure. Asterisks indicates early-late change is significant (* *p* < .05); ✝ indicates main effect of group is significant; and when necessary superscript letters are used to indicate differences between groups, i.e., the same letter is used for any pair when there is a significant difference observed (if bold *p* < 0.01, otherwise *p* < 0.05). | | | | | |

# Supplementary data

## Between group comparison of changes in body mass index and body composition

BMI significantly differed between all three time points [*ŋp^2^* = 0.256; *p* < .001; post hoc results all *p* < .001], see table 3. There was also a week x group interaction [*ŋp^2^* = 0.511; *p* < .001] that revealed that compared with NWL, CWL had a significantly greater reduction in BMI at all three timepoints: baseline and week 2 [CWL: -1.16 ± 0.43 kg/m^2^ (-1.29, -1.03 kg/m^2^); NWL: -0.57 ± 0.43 kg/m^2^ (-0.72, -0.43 kg), *ŋp^2^* = 0.321; *p* < .001]; baseline and week 14 [CWL: -2.68 ± 0.97 kg/m^2^ (-2.98, -2.38 kg/m^2^); NWL: -0.43 ± 0.98 kg/m^2^ (-0.77, -0.09 kg/m^2^), *ŋp^2^* = 0.570; *p* < .001]; and weeks 2 and 14 [CWL: -1.52 ± 0.90 kg/m^2^ (-1.80, -1.24 kg/m^2^); NWL: 0.15 ± 0.90 kg/m^2^ (-0.17, -0.46 kgm^2^), *ŋp^2^* = 0.459; *p* < .001]. There was a significant interaction between week and programme type for BMI [*p* = .04].

On average, FM was higher early in the intervention (week 2 [41.91 ± 7.26 kg (39.35, 44.48 kg)]) compared to late in the intervention (week 14 [40.00 ± 6.99 kg (37.45, 42.56 kg), *ŋp^2^* = 0.157; *p* = .01]). CWL experienced a significant early-late decrease in FM [-3.83 ± 2.14 kg (-4.60, -3.06 kg)] whereas NWL did not [0.01 ± 2.18 kg (-0.79, 0.81 kg), *ŋp^2^* = 0.438; *p* < .001]. The main effect of group was not significant [*ŋp^2^* = 0.052; *p* = .08]. Similarly, for FFM the main effect of week and group and the week x group interaction were not significant [largest *ŋp^2^* = 0.044; smallest *p* = .11].
